# New onset diabetes mellitus and cardiovascular outcomes according to statin intensity in patients after drug-eluting stent implantation in Asian patients

**DOI:** 10.1038/s41598-023-42277-w

**Published:** 2023-09-25

**Authors:** Jaehoon Chung, Hack-Lyoung Kim, Woo-Hyun Lim, Jae-Bing Seo, Joo-Heeg Zo, Myung-A Kim, Sang-Hyun Kim

**Affiliations:** 1 Division of Cardiology, Department of Internal Medicine, National Medical Center, Seoul, Republic of Korea; 2 Division of Cardiology, Department of Internal Medicine, Boramae Medical Center, Seoul National University College of Medicine, 20, Boramae-Ro 5-Gil, Dongjak-Gu, Seoul, 07061 Republic of Korea

**Keywords:** Cardiology, Endocrinology

## Abstract

We investigated the effect of statin intensity on the development of new onset diabetes mellitus (NODM) in Korean patients after percutaneous coronary intervention (PCI). A total of 1013 consecutive patients without diabetes mellitus were retrospectively analyzed. All study patients received high- or moderate-intensity statin (high-intensity statin; 321 [31.7%], moderate-intensity statin; 692 [68.3%]). The primary endpoint was development of NODM, and the secondary one was the composite of cardiac death, non-fatal myocardial infarction, and any revascularization. In 264 pairs (528 patients) of propensity score-matched patient, NODM developed in 34 patients (6.4%) and composite cardiac events occurred in 73 patients (13.8%) during a median follow-up of 36.7 months. The incidence rate of NODM was significantly higher in patients with high-intensity statin than with moderate-intensity statin (8.3% vs. 4.5%, log-rank P = 0.026). The incidence rate of composite events was not significantly different between the two groups (12.5% vs.15.2%, log-rank P = 0.495). The use of high-intensity statins was associated with NODM after adjustment for multiple risk factors (adjusted hazard ratio, 2.18, 95% confidence interval 1.10‒4.51, P = 0.025). High-intensity statin therapy is associated with a higher incidence of NODM, but not with better cardiovascular outcomes, in Korean patients undergoing PCI. A new cholesterol lowering intensity-based approach rather than stain intensity-based approach to the high-risk patients without diabetes mellitus may be helpful in maximal treatment effect without safety concern of NODM in Asian patients.

## Introduction

Coronary heart disease is a leading cause of mortality and morbidity worldwide^1^. The elevated low-density lipoprotein cholesterol (LDL-C) level has been considered one of the major causes of coronary heart disease, and clinical guidelines emphasize LDL-C as the primary target of cholesterol-lowering therapy^2,3^. Statin blocks endogenous synthesis of cholesterol and reduces LDL-C concentration by inhibiting 3-hydroxy-3-methylglutaryl-coenzyme A (HMG-CoA) reductase, which catalyzes the rate-limiting step in cholesterol biosynthesis^4^. In addition, statins inhibit HMG-CoA reductase within endothelial cells, vascular smooth muscle cells, and inflammatory cells, which results in the inhibition of the important signaling pathways of atherosclerosis as pleiotropic effects^5^. Consequently, many previous trials with statins have consistently shown significant reductions in cardiovascular mortality and morbidity^6–9^. Furthermore, high-intensity statin therapy has shown further reductions in cardiovascular events compared with low-intensity or moderate-intensity statin therapy^10^.

However, recent emerging evidence has suggested that statins confer increased risk of developing new-onset diabetes mellitus (NODM)^11–14^. It has been suggested that the risk of developing NODM during statin therapy is associated with statin intensity^12,15,16^. In meta-analysis of data from 5 statin trials, high-intensity statin therapy more increased the incidence of NODM by 12% than moderate-intensity statin therapy^12^.

Clinical lipid guidelines recommend high-intensity statins for the patients with established CVD^2,3^. However, it is well known that Asians achieve more cholesterol reductions and experience more side effects with the same statin intensity than Caucasians^17,18^. Therefore, in Asian countries, it is common for clinicians to use moderate- or low-intensity statin therapy rather than high-intensity statin therapy, even if they are at high risk^19^. In spite of these ethnic differences, most studies on the association between statin use and development of NODM have been conducted in Western countries^13,20–22^. Asian data on the association between statin intensity and NODM in high-risk patients are needed. The data will be very helpful for many clinicians treating Asian patients. Therefore, this study was conducted to investigate the occurrence of NODM and clinical outcomes according to statin intensity in high-risk Korean patients who underwent percutaneous coronary intervention (PCI) for which high-intensity statin is recommended.

## Methods

### Study population

This retrospective, observational, single-center study was conducted at Seoul Boramae hospital in a big city (Seoul, South Korea). Between January 2010 and December 2015, a total of 5080 consecutive patients who received PCI with drug-eluting stents (DES) as well as statin therapy were initially screened. Patients with the following conditions were excluded sequentially: (1) prior history of diabetes mellitus, (2) newly diagnosed diabetes mellitus during the admission for index PCI, (3) loss of clinical follow-up for more than 6 months after PCI, (4) no information on key data for the study analysis such as glucose, glycated hemoglobin and LDL-C levels, and (5) changes in statin intensity during the first year. Finally, medical records of 1014 patients were analyzed in this study. Our study was conducted according to the Declaration of Helsinki. The Institutional Review Board (IRB) of our hospital approved the study protocol (IRB number, 26-2014-66). Informed consent was waived by the IRB due to retrospective study design and to the routine nature of information collected.

### Data collection

Patients’ demographic parameters, underlying disease, smoking status, and concomitant medications were reviewed. Body mass index (BMI) was calculated by dividing body weight in kilograms by the square of the height in meters (kg/m^2^). Previous history of cardiovascular risk factors, including hypertension, dyslipidemia, myocardial infarction, angina, heart failure, ischemic stroke, and smoking status were reviewed. A patient was classified as a current smoker, unless sustained abstinence from cigarettes and/or other tobacco products for at least 12 months. Biochemical parameters, including white blood cell count, hemoglobin, plasma glucose, glycated hemoglobin, total cholesterol, LDL-C, high-density lipoprotein cholesterol, triglyceride, and C-reactive protein, were measured after overnight fasting during index hospitalization. Blood samples for biochemical parameters were collected on the next morning after PCI from patients with ST-elevation myocardial infarction (STEMI) and non-ST-elevation myocardial infarction (NSTEMI) who exhibited unstable vital signs who required emergency PCI. Blood samples for biochemical parameters were collected on the morning of PCI from patients with angina pectoris and NSTEMI with stable vital signs who did not require emergency PCI. Estimated glomerular filtration rate was calculated with the Modification of Diet in Renal Disease Study (MDRD) equation.

### PCI procedures

All PCI procedures were performed in accordance with current procedural guideline^23,24^. All patients received 300 mg loading dose of aspirin and 300 or 600 mg loading dose of clopidogrel unless they had received those antiplatelet agents regularly before the procedures. During the PCI, weight-adjusted intravenous unfractionated heparin was administered to maintain activated clotting time between 250 and 350 s. The choice of DES, usage of glycoprotein IIb/IIIa inhibitors and intravascular imaging devices during the procedure, as well as adjuvant balloon dilatation after stent implantation were at the discretion of the physician. The extent of coronary artery disease was defined as the number of diseased coronary arteries with at least 50% stenosis among the major coronary arteries or their branches sized at least 2 mm in luminal diameter on coronary angiography.

### Statin intensity

The intensity of statin therapy was divided into 2 categories according to the current American College of Cardiology/American Heart Association (ACC/AHA) cholesterol guideline^2^. Statins which lower LDL-C levels equal or more than 50% (Atorvastatin 40‒80 mg, Rosuvastatin 20‒40 mg) were classified as the high-intensity statin group, those with 30% to 49% reduction (Atorvastatin 10‒20 mg, Rosuvastatin 5‒10 mg, Simvastatin 20‒40 mg, Pravastatin 40‒80 mg, and Pitavastatin 2‒4 mg) were classified as the moderate-intensity statin group. The type and dose of statin were prescribed according to clinical guidelines whenever possible, and the final decision was left to the judgement of the attending physician.

### Study outcomes

The primary endpoint of this study was development of NODM during the follow-up period. The secondary endpoint was the major adverse cardiovascular events (MACE), a composite of cardiac death, non-fatal myocardial infarction, and ischemia-driven coronary revascularization. NODM was defined as newly detected glycated hemoglobin A1c (HbA1c) of 6.5% or more, fasting plasma glucose of 126 mg/dL or more, random plasma glucose of 200 mg/dL or more, and medical records of type 2 diabetes mellitus or prescription of one or more antidiabetic medication during the follow-up period. Cardiac death was defined as death from acute coronary syndrome, ventricular arrhythmia, and pump failure. Unexplained sudden death was also considered cardiac death. Myocardial infarction was defined as elevation in cardiac troponin values with at least 1 value above the 99th percentile upper reference limit, with symptoms of myocardial ischemia, new ischemic change of electrocardiography, development of pathologic Q waves, or imaging evidence of myocardial infarction. Ischemia-driven coronary revascularization indicates PCI or coronary artery bypass graft surgery in patients with ≥ 70% diameter stenosis on coronary angiography accompanied by recurrent symptoms, new electrocardiographic changes indicating ischemia, or positive functional study results suggesting ischemia. Clinical follow-up was done every 3–6 months and whenever a clinical event took place. All events were identified by the physician in charge and confirmed by the principal investigator.

### Statistical analysis

The data are presented as the mean ± standard deviation (SD) for continuous variables and as numbers with percentages for categorical variables. Continuous variables were compared using Student’s *t*-test, and categorical variable using Pearson’s chi-square test between the 2 groups. As statin intensity was not randomized, a propensity score was used to adjust for potential selection or predisposition bias. The propensity score was estimated using multiple logistic regression analysis, with all variables shown in Supplementary Table 1. The patients were selected by 1:1 matching without replacement using a greedy algorithm and the nearest available pair matching methods based on propensity score. A caliper width of 0.2 SD of the logit of the estimated propensity score was used for matching. Covariate balance achieved by propensity score-matching was assessed by calculating the absolute standardized differences in covariates between the two groups. After propensity score matching, development of NODM and clinical events were compared with the log-rank test and presented with the Kaplan–Meier survival curves. To estimate hazard ratio (HR) and confidence intervals (CI), cox regression analysis was performed. To determine independent association of intensity of statin and study endpoints, multivariable Cox regression analysis was used. BMI, triglyceride, fasting plasma glucose were included as covariates in the multivariable model to predict the development of NODM. Age, sex, BMI, hypertension, current cigarette smoking status, family history of premature cardiovascular event, and clinical diagnosis at presentation were included as covariates in the multivariable model to predict MACE. All analyses were 2-tailed, and clinical significance was set at *P* < 0.05. All statistical analyses were performed using statistical package SPSS V.22.0 (IBM Co., Armonk, NY, USA) and R programming language V.3.5.3 (R Foundation for Statistical Computing, Vienna, Austria).

## Results

### Baseline clinical characteristics of the study patients

#### Whole study patients

The mean age of the patients in the study was 66.1 ± 11.9 years, and 68.3% were men. A diagnosis of acute coronary syndrome was made in 89% of the study subjects. According to statin intensity, patients were divided into 2 groups: 283 (27.9%) patients received high-intensity statin therapy and 730 (72.1%) patients received moderate-intensity statin therapy. The specific baseline clinical characteristics of the whole study patients according to statin intensity are shown in Supplementary Table 1. Patients with high-intensity statin therapy were younger, taller and heavier than those with moderate-intensity statin therapy. In sex distribution, the proportion of men was higher in the high-intensity statin group than in the moderate-intensity statin group. There were no significant differences in cardiovascular risk factors except current smoking which was more frequent in patients with high-intensity statin therapy than with moderate-intensity statin therapy. For laboratory findings, patients with high-intensity statin therapy showed more unfavorable profiles, including higher blood levels of white blood cell count, total cholesterol and LDL-C, than those with moderate-intensity statin therapy. Clinical diagnosis was significantly different between the 2 groups. The incidence of myocardial infarctions at presentation were higher in the high-intensity statin group. Coronary disease extents were not significantly different between the 2 groups. Prescription rates of cardiovascular medications were similar in the 2 groups. The final mean LDL-C level was significantly lower and percent reduction of most recent LDL-C level was significantly greater in patients with high-intensity statin therapy than with moderate-intensity statin therapy. The detailed values are shown in Supplementary Table 2.

#### Propensity score-matched patients

Propensity score matching was performed to adjust for differences of baseline characteristics. A total of 264 pairs of propensity score-matched patients were analyzed in this study. The p value of Hosmer–Lemeshow goodness of fit for the propensity score model was 0.978. There were no significant differences in the baseline clinical characteristics between the two groups. The baseline clinical characteristics of the propensity score-matched patients are shown in Table 1. The mean age of the study patients was 64.6 ± 12.0 years, and 71.8% were men. The final mean LDL-C level was significantly lower and percent reduction of most recent LDL-C level was significantly greater in patients with high-intensity statin therapy than with moderate-intensity statin therapy. The detailed values are shown in Table 2.

**Table 1:** Baseline characteristics of propensity score matched patients.

Characteristic	High intensity (n = 264)	Moderate intensity (n = 264)	*P* value	Standardized mean difference
Age, years	64.4 ± 11.9	64.9 ± 12.1	0.615	0.043
Men, n (%)	191 (72.3)	188 (71.2)	0.772	0.026
Height, cm	163.1 ± 8.9	162.8 ± 9.5	0.647	0.041
Weight, kg	65.2 ± 11.1	64.6 ± 12.0	0.528	0.054
Body mass index, kg/m^2^	24.4 ± 3.1	24.3 ± 3.5	0.643	0.040
Cardiovascular risk factors, n (%)				
Hypertension	148 (56.1)	150 (56.8)	0.861	0.015
Dyslipidemia	164 (62.1)	172 (65.2)	0.469	0.062
Previous myocardial infarction	13 (4.9)	18 (6.8)	0.355	0.084
Previous angina	15 (5.7)	18 (6.8)	0.590	0.048
Previous heart failure	8 (3.0)	11 (4.2)	0.483	0.068
Previous stroke	18 (6.8)	24 (9.1)	0.335	0.087
Current smoking	100 (37.9)	93 (35.2)	0.527	0.054
Family history of premature CAD	31 (11.7)	30 (11.4)	0.892	0.012
Major laboratory findings				
White blood cell count, per μL	8,380 ± 3,407	8,180 ± 3,145	0.483	0.053
Hemoglobin, g/dL	13.8 ± 1.8	13.8 ± 1.8	0.623	0.042
FPG, mg/dL	101 ± 12	101 ± 13	0.653	0.040
HbA1c, %	5.7 ± 0.4	5.7 ± 0.3	0.670	0.036
eGFR, mL/min/1.73 m^2^	83.8 ± 23.7	83.0 ± 20.9	0.668	0.036
Total cholesterol, mg/dL	185 ± 49	184 ± 43	0.707	0.030
LDL cholesterol, mg/dL	122 ± 34	121 ± 33	0.827	0.017
HDL cholesterol, mg/dL	43 ± 11	44 ± 13	0.219	0.107
Triglyceride, mg/dL	139 ± 274	125 ± 78	0.441	0.051
hs-CRP, mg/dL	0.9 ± 2.2	0.7 ± 1.9	0.415	0.068
Echocardiographic findings				
LVEF, %	61.5 ± 12.4	61.3 ± 12.5	0.883	0.013
Clinical diagnosis			0.866	0.008
Stable angina, n (%)	27 (10.2)	24 (9.1)		
Unstable angina, n (%)	125 (47.3)	125 (47.3)		
NSTEMI, n (%)	58 (22.0)	65 (24.6)		
STEMI, n (%)	54 (20.5)	50 (18.9)		
Diseased coronary artery, n (%)			0.985	0.014
One-vessel disease	70 (26.5)	71 (26.9)		
Two-vessel disease	80 (30.3)	81 (30.7)		
Three-vessel disease	114 (43.2)	112 (42.4)		
Medications, n (%)				
Aspirin	264 (100)	264 (100)	-	0
Clopidogrel	260 (98.5)	260 (98.5)	1.000	0
RAS blocker	185 (70.1)	195 (73.9)	0.333	0.083
Beta blocker	183 (69.3)	191 (72.3)	0.444	0.066
Calcium channel blocker	69 (26.1)	67 (25.4)	0.842	0.017
Nitrate	196 (74.2)	191 (72.3)	0.623	0.043

*CAD* coronary artery disease, *eGFR* estimated glomerular filtration rate, *FPG* fasting plasma glucose, *HDL* high-density lipoprotein, *HbA1c* hemoglobin A1c, *hs-CRP* high-sensitivity C-reactive protein, *LDL* low-density lipoprotein, *LVEF* left ventricular ejection fraction, *NSTEMI* non-ST segment elevation myocardial infarction, *RAS* renin-angiotensin system, *STEMI* ST segment elevation myocardial infarction.

**Table 2:** Low density lipoprotein cholesterol value of propensity score matched patients.

LDL cholesterol, mg/dL	High intensity (n = 264)	Moderate intensity (n = 264)	*P* value
Baseline	121.7 ± 34.2	121.1 ± 32.5	0.827
Final	59.1 ± 15.8	63.2 ± 16.1	0.003
LDL reduction (%)	49.0 ± 15.7	45.6 ± 15.1	0.011
LDL < 70, n (%)	199 (75.4)	180 (68.2)	0.066
LDL < 55, n (%)	112 (42.4)	81 (30.7)	0.005

*LDL* low-density lipoprotein.

### Study outcomes

#### Whole study patients

**Figure 1:**
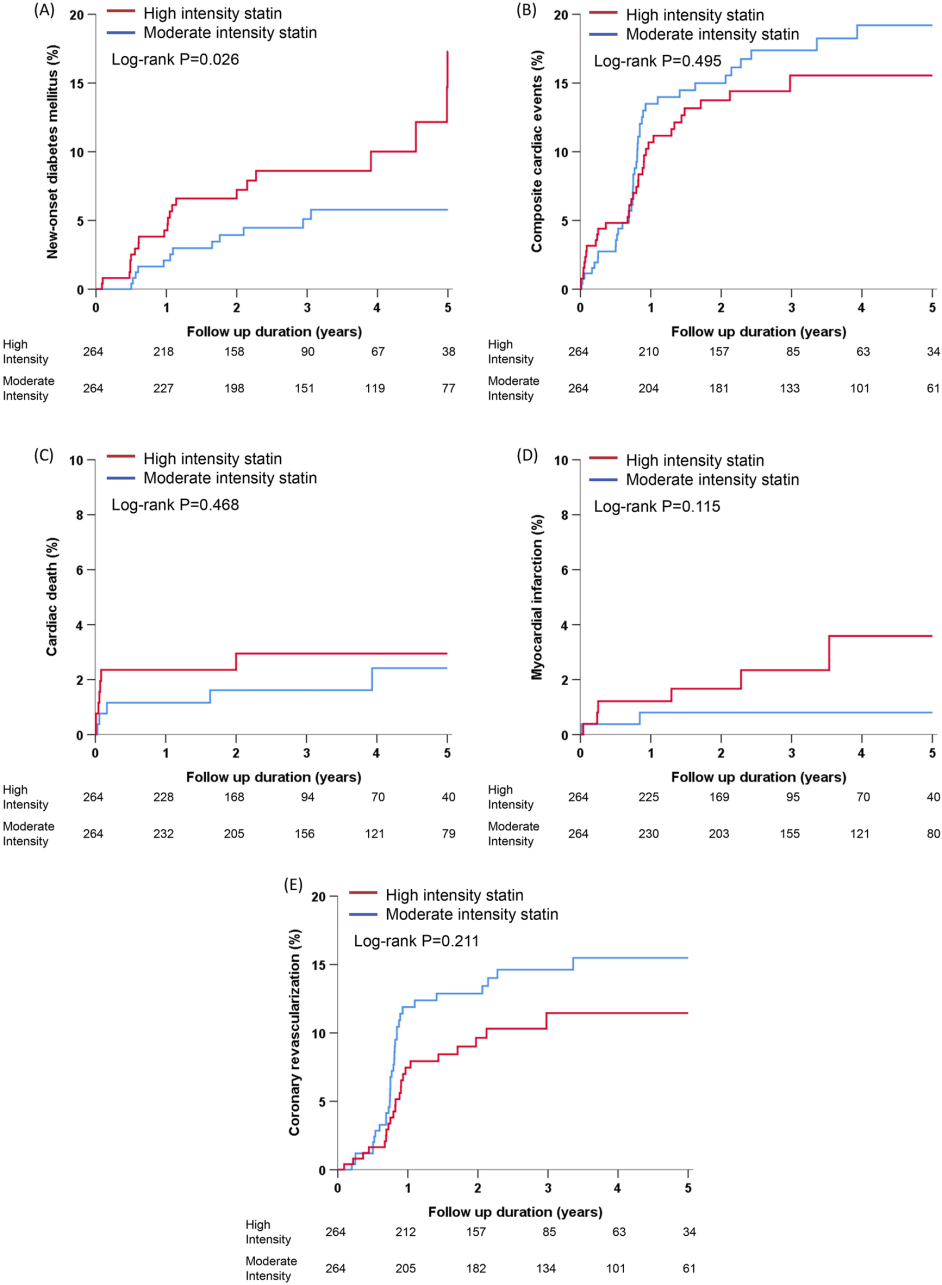
Kaplan–Meier curves for 5-year clinical outcomes according to statin intensity in propensity-score matched population. (**A**) new onset diabetes mellitus, (**B**) composite of cardiac death or ischemia-driven any revascularization, (**C**) cardiac death, (**D**) myocardial infarction, (**E**) any revascularization.

The median follow-up duration was 1098 days (interquartile range: 628–1825 days). During the follow-up period, 61 (6.0%) patients developed NODM as a primary outcome. The incidence of NODM was significantly higher in the high-intensity statin group than in the moderate-intensity statin group (7.8% vs. 5.3%, log-rank *P* = 0.035) (Supplementary Fig. 1A). MACE occurred in 148 (14.6%) patients. The incidence rate of MACE was not different between the high-intensity and moderate-intensity statin groups (13.1% vs. 15.2%, log-rank *P* = 0.660) (Supplementary Fig. 1B). Event rates and Kaplan Meier analysis for each component of MACE (cardiac death, non-fatal myocardial infarction, and repeat revascularization) are presented in Supplementary Table 3 and Fig. 1C–E. The use of high-intensity statins was significantly associated with NODM development after adjusting for multiple potential confounders (high-intensity *vs.* moderate-intensity statin therapy: adjusted HR 1.77, 95% CI 1.05–2.99, *P* = 0.033). However, the incidence of MACE was not associated with statin intensity (adjusted HR 0.91, 95% CI 0.63–1.33, *P* = 0.629) (Supplementary Table 3).

#### Propensity score-matched patients

During the follow-up period, 34 (6.4%) patients developed NODM as a primary outcome. The incidence of NODM was significantly higher in the high-intensity statin group than in the moderate-intensity statin group (8.3% vs. 4.5%, log-rank *P* = 0.026) (Fig. 1A). MACE occurred in 73 (13.8%) patients. The incidence rate of MACE was not different between the high-intensity and moderate-intensity statin groups (12.5% vs. 15.2%, log-rank *P* = 0.495) (Fig. 1B). Event rates and Kaplan Meier analysis for each component of MACE (cardiac death, non-fatal myocardial infarction, and repeat revascularization) are presented in Table 3 and Fig. 1C–E. The use of high-intensity statins was significantly associated with NODM development after adjusting for multiple potential confounders (high-intensity *vs.* moderate-intensity statin therapy: adjusted HR 2.18, 95% CI 1.10–4.51, *P* = 0.025). However, the incidence of MACE was not associated with statin intensity (adjusted HR 0.86, 95% CI 0.54–1.36, *P* = 0.520) (Table 3).

**Table 3:** Clinical outcomes of propensity score matched patients according to statin intensity.

Parameter	High intensity	Moderate intensity	Adjusted HR (95% CI)	*P* value
New-onset diabetes mellitus	22 (8.3%)	12 (4.5%)	2.18 (1.10‒4.51)*	0.025*
MACE	33 (12.5%)	40 (15.2%)	0.86 (0.54‒1.36)^†^	0.520^†^
Cardiac death	7 (2.7%)	5 (1.9%)	1.56 (0.52‒5.04)^†^	0.428^†^
Non-fatal myocardial infarction	6 (2.3%)	2 (0.8%)	3.15 (0.78‒17.64)^†^	0.109^†^
Repeat revascularization	23 (8.7%)	33 (12.5%)	0.72 (0.42‒1.21)^†^	0.218^†^

*HR* hazard ratio, *CI* confidence interval, *MACE* major adverse cardiovascular event.

*Risk for high intensity statin compared to moderate intensity statin. body mass index, baseline fasting plasma glucose, and baseline triglyceride level were included as covariates in multivariable model.

^†^Risk for high intensity statin compared to moderate intensity statin. Age, sex, body mass index, current cigarette smoking status, hypertension, family history of premature coronary artery disease, and clinical diagnosis at presentation were included as covariates in multivariable model.

## Discussion

In this study, we investigated the effect of statin intensity on the development of NODM and composite cardiovascular events in Korean patients who received PCI with DES. The use of high-intensity statins was associated with higher incidence of NODM, than those with moderate-intensity statins. The incidence rate of composite of cardiac death, non-fatal myocardial infarction, and ischemia-driven revascularization was not significantly different between the 2 groups.

Statin decreases intracellular cholesterol synthesis by inhibiting rate-limiting enzymes of cholesterol biosynthesis. By lowering LDL-C and with pleiotropic effects, statins significantly reduce cardiovascular mortality and morbidity^6–9^. However, there is concern about development of NODM associated with statin use. In the Anglo Scandinavian Cardiac Outcomes Trial-Lipid Lowering Arm (ASCOT-LLA) trial, treatment with atorvastatin 10 mg numerically increased risk of NODM (HR 1.15, 95% CI 0.91–1.44), albeit without statistical significance^20^. In the Stroke Prevention by Aggressive Reduction in Cholesterol Levels (SPARCL) trial, NODM developed much more frequently in patients with atorvastatin 80 mg than with placebo (8.71% vs. 6.06%, adjusted HR 1.37, 95% CI 1.08–1.75). In the analysis of Justification for Use of statins in Prevention, an Intervention Trial Evaluating Rosuvastatin (JUPITER) trial, rosuvastatin therapy more increased risk of developing NODM than placebo especially in patients with risk factors for diabetes mellitus (HR 1.28, 95% CI 1.07–1.54)^25^. Sattar et al.^14^. conducted meta-analysis to figure out relationships between statin use and development of NODM, and concluded that statin was associated with slightly increased risk of developing NODM (HR 1.09, 95% CI 1.02–1.17). Furthermore, along with our study results, several previous studies have shown that high-intensity statin therapy more increased risk of NODM than moderate-intensity statin therapy^15,26,27^.

There are racial differences in responses to statin. Plasma exposure to rosuvastatin and its metabolites was significantly higher in Asians than in Caucasians^28^. In an efficacy study with Japanese subjects, 5 mg of rosuvastatin lowered LDL-C by 52.7% than baseline^29^. On the other hand, in Western trails, 5 mg of rosuvastatin lowed LDL by approximately 40%, and 20 mg of rosuvastatin was needed to achieve 52% reduction in LDL-C^30,31^. The magnitude of LDL-C reduction with low- to moderate-intensity statin therapy varies in many clinical outcomes, not simply in laboratory results. In the Management of Elevated Cholesterol in the Primary Prevention Group of Adult Japanese (MEGA) study conducted in Japan, even low-intensity statin therapy significantly reduced cardiovascular outcome (HR 0.67, 95% CI 0.49‒0.91)^32^. High-intensity statin therapy not only reduces cardiovascular events, but also increases side effects, such as statin induced myopathy, and elevation of liver enzyme^15,33,34^. In line with these findings, other studies also demonstrated that Asians achieved more cholesterol reductions and experienced more side effects than Caucasians after receiving the same intensity statin therapy^17,18^. These results suggest that special attention must be paid to the efficacy-side effect ratio for specific statin treatment strategy in Asians.

### Clinical implications

The benefits of statins outweigh numerous side effects, and current clinical guidelines recommend high-intensity statin therapy in patients at high risk, regardless of the presence of diabetes mellitus^2,3^. However, diabetes mellitus increases the risk of cardiovascular disease and other complications, such as retinopathy, nephropathy, neuropathy, diabetic foot, and dementia, during a long-term follow-up^35–39^. Therefore, we consider appropriate lipid lowering therapy to have the best clinical prognosis with minimal adverse effects, including NODM development with life-long statin therapy. In recent study by Abbasi et al., showed that high intensity statin increased insulin resistance^40^. And, study by Dormuth et al. showed that high intensity statin was associated with 15% increased risk of development of NODM compared with lower potency statins^41^. Similar to previous study, in this study, high-intensity statin therapy was significantly associated with higher risk of NODM than moderate-intensity statin therapy in very-high-risk Korean patients who had undergone PCI. Furthermore, high-intensity statin therapy was not more beneficial than moderate-intensity statin therapy in terms of the prevention of cardiovascular events, including cardiac death, non-fatal myocardial infarction, and ischemia-driven revascularization. Although, statin is known to have pleiotropic effects such as anti-inflammatory effects in addition to LDL-C lowering effect, there was no statistically significant difference in clinical events according to statin intensity when LDL-C was lowered to a similar level in this study. It is obvious that the lower LDL-C, the lower CVD event for both primary and secondary prevention^42^. In recent study, the addition of ezetimibe to statin significantly lowered LDL-C level and CVD events without significant increase in side effects^43^. Similarly, recent clinical trials with proprotein convertase subtilisin/Kexin 9 (PCSK9) monoclonal antibodies reported no increase in NODM risk at very low LDL-C levels, it is conceivable that the low LDL-C level is not associated with NODM development^44^. Current cholesterol clinical practice guidelines recommend using maximally tolerated statin first, followed by adding ezetimibe or PCSK9 inhibitor^2,3^. However, in this study, high intensity statin was associated with significant increase of NODM in Asian patients. Therefore, we should consider potent cholesterol lowering therapy rather than high intensity statin therapy to very-high-risk Asian patients who do not have diabetes mellitus before statin therapy. If patients cannot reach therapeutic goal with moderate intensity statin therapy, we are able to add ezetimibe or PCSK9 inhibitor to moderate intensity statin therapy rather than increasing statin dose to reduce risk of NODM. As previously described, there is a racial difference in efficacy and safety by statin therapy. Due to differences in pharmacodynamics and pharmacokinetics of statins, adjustment of cholesterol lowering therapy rather than increase statin intensity might be helpful to increase efficacy and safety in Asian patients.

### Study limitations

There are several limitations in this study. First, this is observational study, so the results may have been subjected to bias, even though propensity score matching method was used and multiple potential variables were adjusted in the analysis. Second, all subjects in this study were Korean, and ethnicity may have affected the risk of developing NODM. Thus, generalization of our study results to other ethnic groups may be difficult. Third, the incidence of cardiovascular events was relatively lower than expected. One plausible explanation is that all the diabetic patients were excluded in the analysis. In addition, a relatively well controlled LDL-C level may have lowered the patients’ risk of developing cardiovascular events. Fourth, although patients whose satin intensity changed within the first 1 year were excluded in the study, there is a possibility that statin intensity was altered in some patients during the follow-up period after 1 year; however, it was not adequately reflected in this study. Fifth, it is not clear what type and dose of statins the patients had been using prior to the study, or whether the patients had never used statins. An individual history of statin use prior to the study may also have influenced the physician's decision to select the appropriate statin type and dosage. In fact, in this study, it is unclear whether it was an effect of previous statin use, but the LDL-C reduction did not exceed 50% compared to baseline in patients who received high-intensity statin therapy. This suggests that the reduction in LDL-C was less than expected, compared to the 45.6% LDL-C reduction in the moderate intensity statin group. Duration of statin use is also an important factor in affecting the incidence of NODM, so the duration of each subject's statin use before the study may have influenced the results. Based on the LDL-C reduction rate, patients prescribed high intensity statins were more likely to have taken statins previously than those prescribed moderate intensity statins. Therefore, the effects of previously used statins may not have been adjusted, which may have affected the study results. Sixth, lifestyle factors such as diet and exercise are also important factors contributing to the development of NODM. In crude patients, the high intensity statin group showed higher smoking rate compared with the moderate statin group. Since, lifestyle factors were not assessed in this study, lifestyle factor could have influenced study results. Seventh, contemporary drug eluting stent and wide usage of intracoronary imaging and optimal stent technique could have influenced development of MACE. Eighth, side effects related to statin use other than diabetes were not assessed in this study. Contrary to expectations, the occurrence of cardiac death and myocardial infarction was higher in the high-intensity statin group. It is speculated that the cardiac event occurred when the patient arbitrarily stopped taking the statin due to side effects. Finally, it is unknown whether the lowered LDL-C level itself, rather than statin intensity, caused NODM. However, as previously described, recent clinical trials with PCSK9 inhibitor reported no increase in NODM risk at very low LDL-C levels, it is conceivable that the low LDL-C level is not associated with NODM development^44^.

## Conclusions

High-intensity statin therapy, rather than moderate-intensity statin therapy, is associated with a higher incidence of NODM and was not associated with better cardiovascular outcomes in patients who received PCI. A new cholesterol lowering intensity-based approach to the treatment of high-risk patients should be considered in achieving the maximal treatment effect without safety concern of NODM. More detailed guidelines for statin therapy are needed to maintain efficacy and to increase safety for Asian populations. Larger-scaled, prospective, randomized controlled trials may be helpful in clarifying the effect and safety of a new cholesterol lowering intensity-based approach rather than statin intensity-based approach for the treatment of high-risk patients without diabetes mellitus.

### Supplementary Information

Supplementary Information.

## Data Availability

The data generated or analyzed during the study are included in this manuscript. However, the dataset is available from the corresponding author on reasonable request.

## References

[1] Virani SS (2020). Heart disease and stroke statistics-2020 update: A report from the American Heart Association. Circulation.

[2] Grundy SM (2019). 2018 AHA/ACC/AACVPR/AAPA/ABC/ACPM/ADA/AGS/APhA/ASPC/NLA/PCNA Guideline on the Management of Blood Cholesterol: A report of the American College of Cardiology/American Heart Association Task Force on clinical practice guidelines. Circulation.

[3] Mach F (2020). 2019 ESC/EAS Guidelines for the management of dyslipidaemias: Lipid modification to reduce cardiovascular risk. Eur. Heart J..

[4] Grundy SM (1988). HMG-CoA reductase inhibitors for treatment of hypercholesterolemia. N. Engl. J. Med..

[5] Gibson CM (2009). Effect of intensive statin therapy on clinical outcomes among patients undergoing percutaneous coronary intervention for acute coronary syndrome. PCI-PROVE IT: A PROVE IT-TIMI 22 (pravastatin or atorvastatin evaluation and infection therapy-thrombolysis in myocardial infarction 22) substudy. J. Am. Coll. Cardiol..

[6] Group, S. S. S. S (1994). Randomised trial of cholesterol lowering in 4444 patients with coronary heart disease: The Scandinavian Simvastatin Survival Study (4S). Lancet.

[7] Athyros VG (2002). Treatment with atorvastatin to the National Cholesterol Educational Program goal versus 'usual' care in secondary coronary heart disease prevention. The GREek Atorvastatin and Coronary-heart-disease Evaluation (GREACE) study. Curr. Med. Res. Opin..

[8] Sacks FM (1996). The effect of pravastatin on coronary events after myocardial infarction in patients with average cholesterol levels. Cholesterol and Recurrent Events Trial investigators. N. Engl. J. Med..

[9] Schwartz GG (2001). Effects of atorvastatin on early recurrent ischemic events in acute coronary syndromes: The MIRACL study: A randomized controlled trial. JAMA.

[10] Cholesterol Treatment Trialists (2010). Efficacy and safety of more intensive lowering of LDL cholesterol: A meta-analysis of data from 170,000 participants in 26 randomised trials. Lancet.

[11] Carter AA (2013). Risk of incident diabetes among patients treated with statins: Population based study. BMJ.

[12] Preiss D (2011). Risk of incident diabetes with intensive-dose compared with moderate-dose statin therapy: A meta-analysis. JAMA.

[13] Ridker PM, Pradhan A, MacFadyen JG, Libby P, Glynn RJ (2012). Cardiovascular benefits and diabetes risks of statin therapy in primary prevention: An analysis from the JUPITER trial. Lancet.

[14] Sattar N (2010). Statins and risk of incident diabetes: A collaborative meta-analysis of randomised statin trials. Lancet.

[15] Study of the Effectiveness of Additional Reductions in C (2010). Intensive lowering of LDL cholesterol with 80 mg versus 20 mg simvastatin daily in 12,064 survivors of myocardial infarction: A double-blind randomised trial. Lancet.

[16] Waters DD (2011). Predictors of new-onset diabetes in patients treated with atorvastatin: Results from 3 large randomized clinical trials. J. Am. Coll. Cardiol..

[17] Group, H. T. C (2013). HPS2-THRIVE randomized placebo-controlled trial in 25 673 high-risk patients of ER niacin/laropiprant: Trial design, pre-specified muscle and liver outcomes, and reasons for stopping study treatment. Eur. Heart J..

[18] Liao JK (2007). Safety and efficacy of statins in Asians. Am. J. Cardiol..

[19] Nagar SP (2018). Treatment patterns, statin intolerance, and subsequent cardiovascular events among Japanese patients with high cardiovascular risk initiating statin therapy. Circ. J..

[20] Sever PS (2003). Prevention of coronary and stroke events with atorvastatin in hypertensive patients who have average or lower-than-average cholesterol concentrations, in the Anglo-Scandinavian Cardiac Outcomes Trial-Lipid Lowering Arm (ASCOT-LLA): A multicentre randomised controlled trial. Lancet.

[21] Collins R (2003). MRC/BHF Heart Protection Study of cholesterol-lowering with simvastatin in 5963 people with diabetes: A randomised placebo-controlled trial. Lancet.

[22] Shepherd J (2002). Pravastatin in elderly individuals at risk of vascular disease (PROSPER): A randomised controlled trial. Lancet.

[23] Authors TF (2014). ESC/EACTS Guidelines on myocardial revascularization: The Task Force on Myocardial Revascularization of the European Society of Cardiology (ESC) and the European Association for Cardio-Thoracic Surgery (EACTS)Developed with the special contribution of the European Association of Percutaneous Cardiovascular Interventions (EAPCI). Eur. Heart J..

[24] Patel MR, Dehmer GJ, Hirshfeld JW, Smith PK, Spertus JA (2012). ACCF/SCAI/STS/AATS/AHA/ASNC/HFSA/SCCT 2012 Appropriate use criteria for coronary revascularization focused update: A report of the American College of Cardiology Foundation Appropriate Use Criteria Task Force, Society for Cardiovascular Angiography and Interventions, Society of Thoracic Surgeons, American Association for Thoracic Surgery, American Heart Association, American Society of Nuclear Cardiology, and the Society of Cardiovascular Computed Tomography. J. Am. Coll. Cardiol..

[25] Ridker PM (2008). Rosuvastatin to prevent vascular events in men and women with elevated C-reactive protein. N. Engl. J. Med..

[26] Pedersen TR (2005). High-dose atorvastatin vs usual-dose simvastatin for secondary prevention after myocardial infarction: The IDEAL study: A randomized controlled trial. JAMA.

[27] Cannon CP (2004). Intensive versus moderate lipid lowering with statins after acute coronary syndromes. N. Engl. J. Med..

[28] Lee E (2005). Rosuvastatin pharmacokinetics and pharmacogenetics in white and Asian subjects residing in the same environment. Clin. Pharmacol. Ther..

[29] Saito Y, Goto Y, Dane A, Strutt K, Raza A (2003). Randomized dose-response study of rosuvastatin in Japanese patients with hypercholesterolemia. J. Atheroscler. Thromb..

[30] Schneck DW (2003). Comparative effects of rosuvastatin and atorvastatin across their dose ranges in patients with hypercholesterolemia and without active arterial disease. Am. J. Cardiol..

[31] Schwartz GG (2004). Efficacy and safety of rosuvastatin and atorvastatin in patients with hypercholesterolemia and a high risk of coronary heart disease: A randomized, controlled trial. Am. Heart J..

[32] Nakamura H (2006). Primary prevention of cardiovascular disease with pravastatin in Japan (MEGA Study): A prospective randomised controlled trial. Lancet.

[33] Dujovne CA (1991). Expanded clinical evaluation of lovastatin (EXCEL) study results: IV. Additional perspectives on the tolerability of lovastatin. Am. J. Med..

[34] Heart Protection Study Collaborative, G (2002). MRC/BHF Heart Protection Study of cholesterol lowering with simvastatin in 20,536 high-risk individuals: A randomised placebo-controlled trial. Lancet.

[35] Afkarian M (2016). clinical manifestations of kidney disease among US adults with diabetes, 1988–2014. JAMA.

[36] Solomon SD (2017). Diabetic retinopathy: A position statement by the American Diabetes Association. Diabetes Care.

[37] Ismail-Beigi F (2010). Effect of intensive treatment of hyperglycaemia on microvascular outcomes in type 2 diabetes: An analysis of the ACCORD randomised trial. Lancet.

[38] Pop-Busui R (2017). Diabetic neuropathy: A position statement by the American Diabetes Association. Diabetes Care.

[39] Biessels GJ, Despa F (2018). Cognitive decline and dementia in diabetes mellitus: Mechanisms and clinical implications. Nat. Rev. Endocrinol..

[40] Abbasi F (2021). statins are associated with increased insulin resistance and secretion. Arterioscler. Thromb. Vasc. Biol..

[41] Dormuth CR (2014). Higher potency statins and the risk of new diabetes: Multicentre, observational study of administrative databases. BMJ.

[42] Sabatine MS (2016). Advances in the treatment of dyslipidemia. Cleve Clin. J. Med..

[43] Cannon CP (2015). Ezetimibe added to statin therapy after acute coronary syndromes. N. Engl. J. Med..

[44] Colhoun HM (2016). No effect of PCSK9 inhibitor alirocumab on the incidence of diabetes in a pooled analysis from 10 ODYSSEY Phase 3 studies. Eur. Heart J..

