# Supplementary material for: New onset diabetes mellitus and cardiovascular outcomes according to statin intensity in patients after drug-eluting stent implantation in Asian patients
Source: Sci Rep. 2023 Sep 25;13:16061. doi: 10.1038/s41598-023-42277-w (PMC10520042; doi:10.1038/s41598-023-42277-w)
Supplement: Supplementary file 1 — Supplementary Information. [file 41598_2023_42277_MOESM1_ESM.docx]

**Supplementary table 1. Baseline characteristics of crude patients**

| **Characteristic** | **High intensity**  **(n = 283)** | **Moderate intensity**  **(n =731)** | ***P* value** | **Standardized mean difference** |
| --- | --- | --- | --- | --- |
| Age, years | 63.9 ± 12.4 | 66.9 ± 11.6 | <0.001 | 0.249 |
| Men, n (%) | 207 (73.1) | 485 (66.3) | 0.037 | 0.153 |
| Height, cm | 163.5 ± 8.9 | 161.8 ± 9.4 | 0.009 | 0.191 |
| Weight, kg | 65.9 ± 11.8 | 63.6 ± 11.5 | 0.004 | 0.198 |
| Body mass index, kg/m^2^ | 24.5 ± 3.3 | 24.2 ± 3.4 | 0.147 | 0.104 |
| Cardiovascular risk factors, n (%) |  |  |  |  |
| Hypertension | 155 (54.8) | 431 (59.0) | 0.226 | 0.084 |
| Dyslipidemia | 177 (62.5) | 453 (62.0) | 0.866 | 0.012 |
| Previous myocardial infarction | 15 (5.3) | 52 (7.1) | 0.297 | 0.081 |
| Previous angina | 17 (6.0) | 55 (7.5) | 0.399 | 0.064 |
| Previous heart failure | 8 (2.8) | 30 (4.1) | 0.337 | 0.077 |
| Previous stroke | 21 (7.4) | 62 (8.5) | 0.580 | 0.087 |
| Current smoking | 111 (39.2) | 226 (30.9) | 0.012 | 0.170 |
| Family history of premature CAD | 32 (11.3) | 70 (9.6) | 0.411 | 0.055 |
| Major laboratory findings |  |  |  |  |
| White blood cell count, per *μ*L | 8,718 ± 3,777 | 7,703 ± 2,870 | <0.001 | 0.269 |
| Hemoglobin, g/dL | 13.9 ± 1.8 | 13.5 ± 1.8 | 0.002 | 0.211 |
| FPG, mg/dL | 101 ± 12 | 102 ± 12 | 0.109 | 0.111 |
| HbA1c, % | 5.7 ± 0.4 | 5.7 ± 0.4 | 0.448 | 0.055 |
| eGFR, mL/min/1.73m^2^ | 83.7 ± 23.5 | 81.6 ± 21.7 | 0.189 | 0.087 |
| Total cholesterol, mg/dL | 189 ± 50 | 170 ± 41 | <0.001 | 0.372 |
| LDL cholesterol, mg/dL | 125 ± 36 | 114 ± 30 | <0.001 | 0.298 |
| HDL cholesterol, mg/dL | 43 ± 11 | 43 ± 12 | 0.934 | 0.006 |
| Triglyceride, mg/dL | 138 ± 266 | 119 ± 69 | 0.073 | 0.072 |
| hs-CRP, mg/dL | 0.8 ± 2.2 | 0.9 ± 2.7 | 0.437 | 0.045 |
| Echocardiographic findings |  |  |  |  |
| LVEF, % | 61.5 ± 12.4 | 60.6 ± 13.3 | 0.322 | 0.073 |
| Clinical diagnosis |  |  | <0.001 | 0.296 |
| Stable angina, n (%) | 27 (9.5) | 93 (12.7) |  |  |
| Unstable angina, n (%) | 128 (45.2) | 418 (57.2) |  |  |
| NSTEMI, n (%) | 63 (22.3) | 115 (15.7) |  |  |
| STEMI, n (%) | 65 (23.0) | 105 (14.4) |  |  |
| Diseased coronary artery, n (%) |  |  | 0.104 | 0.117 |
| One-vessel disease | 72 (25.4) | 203 (27.8) |  |  |
| Two-vessel disease | 83 (29.3) | 250 (34.2) |  |  |
| Three-vessel disease | 128 (45.2) | 278 (38.0) |  |  |
| Medications, n (%) |  |  |  |  |
| Aspirin | 283 (100) | 730 (99.9) | 0.534 | 0 |
| Clopidogrel | 279 (98.6) | 725 (99.2) | 0.392 | 0.050 |
| RAS blocker | 199 (70.3) | 534 (73.1) | 0.383 | 0.060 |
| Beta blocker | 196 (69.3) | 530 (72.5) | 0.304 | 0.070 |
| Calcium channel blocker | 71 (25.1) | 221 (30.2) | 0.105 | 0.118 |
| Nitrate | 210 (74.2) | 512 (70.0) | 0.189 | 0.095 |
| CAD, coronary artery disease; eGFR, estimated glomerular filtration rate; FPG, fasting plasma glucose; HDL, high-density lipoprotein; HbA1c, hemoglobin A1c; hs-CRP, high-sensitivity C-reactive protein; LDL, low-density lipoprotein; LVEF, left ventricular ejection fraction; NSTEMI, non-ST segment elevation myocardial infarction; RAS, renin-angiotensin system; STEMI, ST segment elevation myocardial infarction. | | | | |

**Supplementary table 2. Low density lipoprotein cholesterol value of crude patients**

| **LDL cholesterol, mg/dL** | **High intensity**  **(n = 283)** | **Moderate intensity**  **(n = 731)** | ***P* value** |
| --- | --- | --- | --- |
| Baseline | 125 ± 36 | 114 ± 30 | <0.001 |
| Final | 60 ± 16 | 63 ± 17 | 0.011 |
| LDL reduction (%) | 49.4 ± 15.7 | 42.5 ± 16.3 | <0.001 |
| LDL < 70, n (%) | 211 (74.6) | 497 (68.0) | 0.041 |
| LDL < 55, n (%) | 116 (41.0) | 230 (31.5) | 0.004 |
| LDL, low-density lipoprotein. | | | |

**Supplementary table 3. Clinical outcomes of crude patients according to statin intensity**

| **Parameter** | **High intensity** | **Moderate intensity** | **Adjusted HR**  **(95% CI)** | ***P* value** |
| --- | --- | --- | --- | --- |
| New-onset diabetes mellitus | 22 (7.8%) | 39 (5.3%) | 1.77 (1.05‒2.99)***** | 0.033***** |
| MACE | 37 (13.1%) | 111 (15.2%) | 0.91 (0.63‒1.33)^†^ | 0.629^†^ |
| Cardiac death | 10 (3.5%) | 17 (2.3%) | 1.47 (0.67‒3.24)^†^ | 0.339^†^ |
| Non-fatal myocardial infarction | 7 (2.5%) | 7 (1.0%) | 2.50 (0.86‒7.27)^†^ | 0.092^†^ |
| Repeat revascularization | 24 (8.5%) | 92 (12.6%) | 0.74 (0.47‒1.16)^†^ | 0.191^†^ |
| *****Risk for high intensity statin compared to moderate intensity statin. body mass index, baseline fasting plasma glucose, and baseline triglyceride level were included as covariates in multivariable model. ^†^Risk for high intensity statin compared to moderate intensity statin. Age, sex, body mass index, current cigarette smoking status, hypertension, family history of premature coronary artery disease, and clinical diagnosis at presentation were included as covariates in multivariable model. HR, hazard ratio; CI, confidence interval. MACE, major adverse cardiovascular event. | | | | |


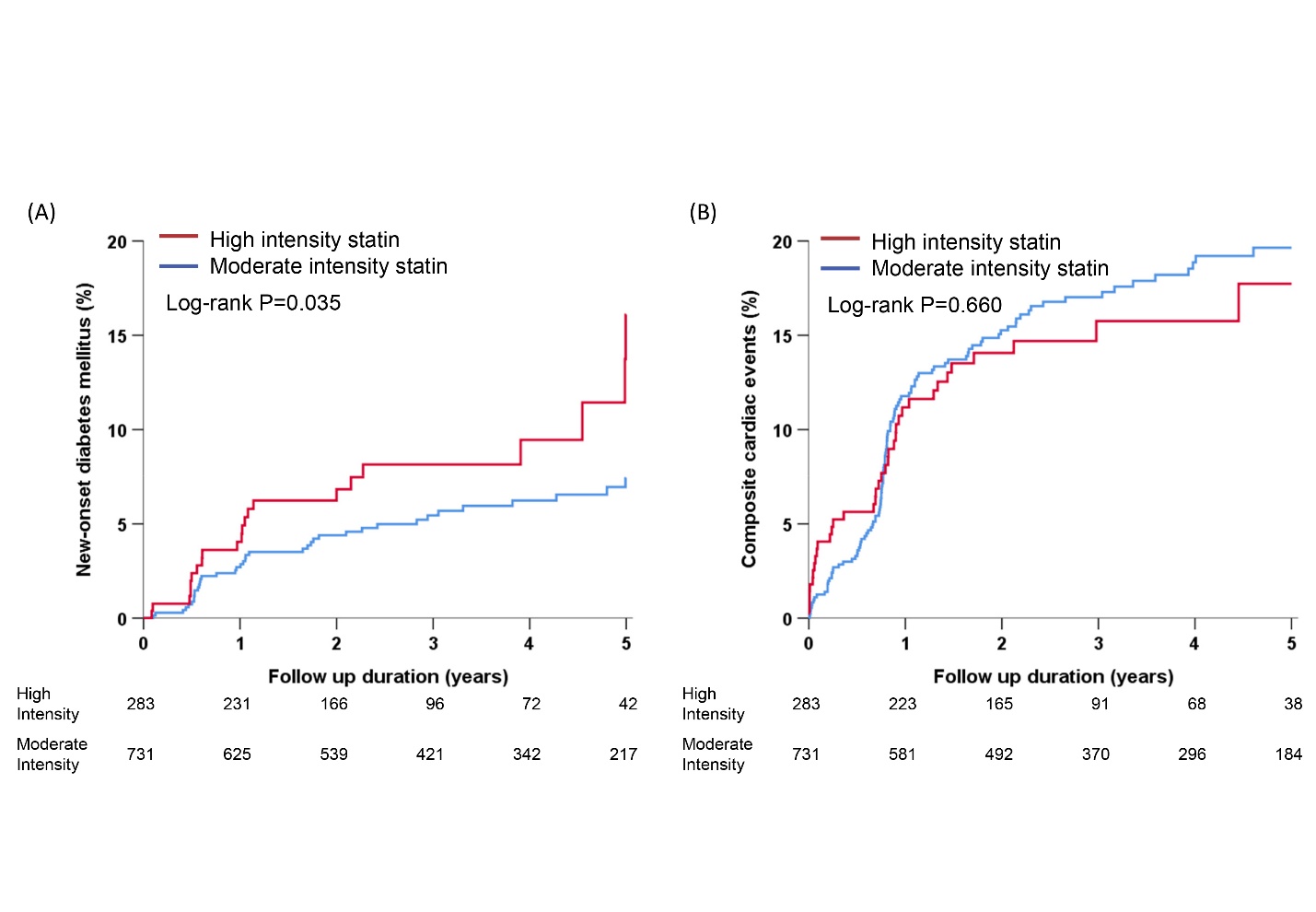

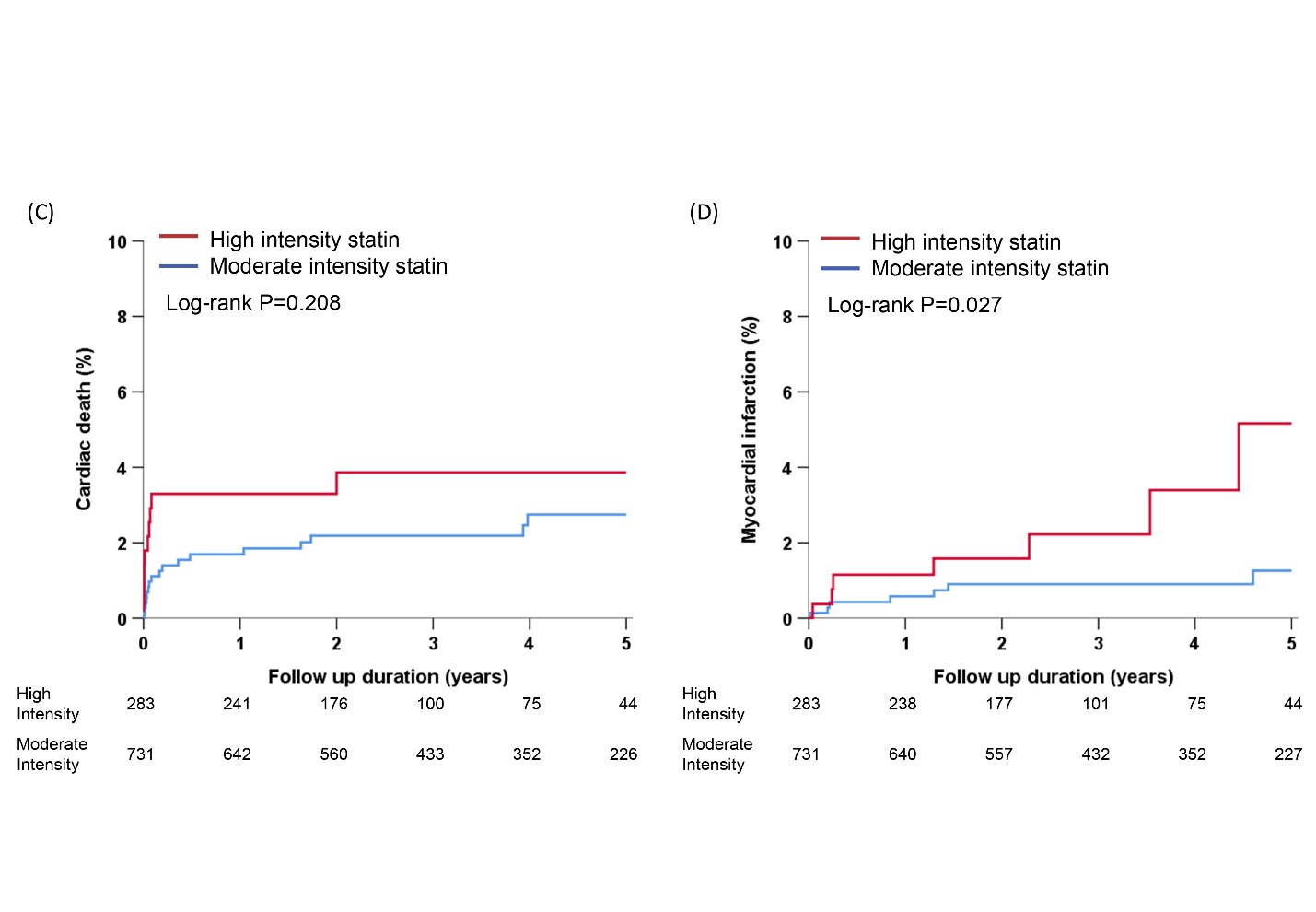

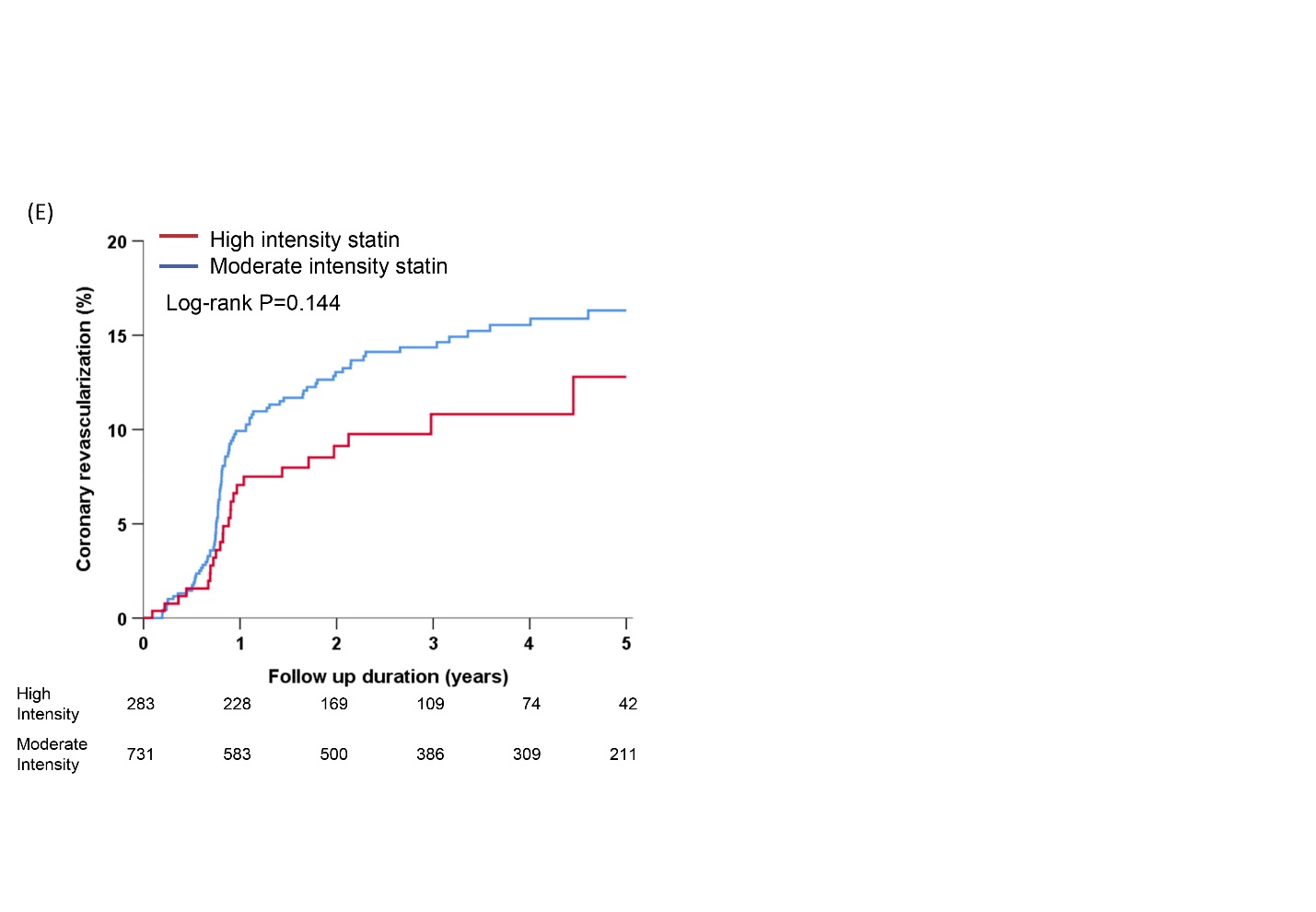


**Figure legend**

**Supplementary Fig. 1. Kaplan-Meier curves for 5-year clinical outcomes according to statin intensity in crude population**

(A) new onset diabetes mellitus, (B) composite of cardiac death or ischemia-driven any revascularization, (C) cardiac death, (D) myocardial infarction, (E) any revascularization.
